# GEP-EpiSeeker: a gene expression programming-based method for epistatic interaction detection in genome-wide association studies

**DOI:** 10.1186/s12864-021-08207-8

**Published:** 2021-12-20

**Authors:** Yu Zhong Peng, Yanmei Lin, Yiran Huang, Ying Li, Guangsheng Luo, Jianping Liao

**Affiliations:** 1 School of Computer & Information Engineering, Nanning Normal University, Nanning, 530001 China; 2 School of Computer science, Fudan University, Shanghai, 200433 China; 3 School of Computer and Electronics and Information, Guangxi Key Laboratory of Multimedia Communications and Network Technology, Guangxi University, Nanning, 530004 China

**Keywords:** Gene Expression Programming, Epistatic Interactions, Epistasis Analysis, Single Nucleotide Polymorphisms, Evolutionary Algorithm

## Abstract

**Background:**

Identification of epistatic interactions provides a systematic way for exploring associations among different single nucleotide polymorphism (SNP) and complex diseases. Although considerable progress has been made in epistasis detection, efficiently and accurately identifying epistatic interactions remains a challenge due to the intensive growth of measuring SNP combinations.

**Results:**

In this work, we formulate the detection of epistatic interactions by a combinational optimization problem, and propose a novel evolutionary-based framework, called GEP-EpiSeeker, to detect epistatic interactions using Gene Expression Programming. In GEP-EpiSeeker, we propose several tailor-made chromosome rules to describe SNP combinations, and incorporate Bayesian network-based fitness evaluation into the evolution of tailor-made chromosomes to find suspected SNP combinations, and adopt the Chi-square test to identify optimal solutions from suspected SNP combinations. Moreover, to improve the convergence and accuracy of the algorithm, we design two genetic operators with multiple and adjacent mutations and an adaptive genetic manipulation method with fuzzy control to efficiently manipulate the evolution of tailor-made chromosomes. We compared GEP-EpiSeeker with state-of-the-art methods including BEAM, BOOST, AntEpiSeeker, MACOED, and EACO in terms of power, recall, precision and *F*1-score on the GWAS datasets of 12 DME disease models and 10 DNME disease models. Our experimental results show that GEP-EpiSeeker outperforms comparative methods.

**Conclusions:**

Here we presented a novel method named GEP-EpiSeeker, based on the Gene Expression Programming algorithm, to identify epistatic interactions in Genome-wide Association Studies. The results indicate that GEP-EpiSeeker could be a promising alternative to the existing methods in epistasis detection and will provide a new way for accurately identifying epistasis.

## Introduction

Genome-wide association studies (GWAS) aim at identifying associations between Single Nucleotide Polymorphism (SNP) and disease, which has been an important way for identifying the genetic basis of diseases in the last decade [1–11].

GWAS is capable of finding single-locus SNP that is related to disease trait [7]. Great progress has been made in identifying single-locus SNP that is the genetic causes of diseases such as Mendelian diseases and diabetes, however, detecting causative loci for complex diseases is more complicated [3, 5, 6, 12]. Complex diseases are often caused by complicated effects of multi-locus SNPs, such as diabetes, rheumatoid arthritis and hypertension [6, 7, 13]. Some SNPs influence the complex disease traits and dominate the effect of other SNPs when interacting with each other [6, 7, 12]. In GWAS, the relation of an SNP influencing the effect of another SNP is described as epistasis [7, 12, 14]. Many studies have shown that epistasis exists in SNP interactions and plays an important role in human diseases [7, 15].

With the rapid development of high-throughput genotyping and sequencing technologies, it is an enormous challenge to analyze the epistatic associations between disease and millions of SNPs in GWAS. Recently, several epistatic interaction detection methods have been designed for efficiently detecting epistasis. These efforts can be divided into three types [3, 6, 13, 14, 16–21] : (1) exhaustive search method, (2) stochastic search method, and (3) heuristic search method.

Exhaustive search method evaluates all possible multi-locus SNP combinations to detect the associations between disease and SNPs. Therefore, exhaustive search methods can produce stable and global optimum solutions. Some exhaustive search methods, such as MDR [22, 23], BOOST [24], TEAM [25], ESMO [6], have been proposed. Exhaustive search is a straightforward search strategy, but it may require huge computational resources and consume too much time as the size of SNP combinations exponentially grows.

Stochastic search-based identifies SNP-SNP interactions by random sampling [26, 27]. BEAM (Bayesian Epistasis Association Mapping) [27] is an example. BEAM searches and categorizes disease-associated SNP interactions via posterior probabilities of the suspected candidate SNPs. Tang et al. [28] constructed a Gibbs sampling approach for identifying epistatic interactions. Jiang et al. [29] presented a stochastic method called epiForest to detect epistatic interactions using random forest. Although random sampling significantly reduces search space and accelerates the detection of SNP interactions, the performance of stochastic search relies on the random sampling elements.

Heuristic search [3, 7, 12, 14, 30] adopts an approximate search strategy, which guides the search of epistatic interactions by heuristic information. For example, Wang et al. [30] proposed a two-stage heuristic ant colony optimization (ACO) algorithm named AntEpiSeeker to detect epistatic interactions. AntEpiSeeker uses an ant colony optimization search to find disease-associated SNPs. Wan et al. [31] developed SNPRuler to detect epistatic interactions utilizing prediction rule learning. Jing et al. [3] presented a multi-objective optimization heuristic method named MACOED, which complementarily combines the logistical regression and Bayesian network to identify epistatic interactions. Yuan et al . [7] designed a multi-objective ACO-based method named FAACOSE. FAACOSE combines multi-objective optimization functions with an adaptive ant colony optimization algorithm to search epistatic interactions. Sun et al . [14] proposed an ACO-based method named EACO for identifying epistatic interactions by incorporating heuristic information multi-SURF(Spatially Uniform ReliefF) into ant-decision rules.

Recently, in addition to the ACO-based algorithm, some other evolutionary methods have also been adopted for the heuristic search of disease-associated SNPs [15, 26, 32]. For example, Yang et al . [32] proposed a Genetic algorithm-based hybrid algorithm, which is named genetic ensemble (GE). GE combines an ensemble of classifiers with a multi-objective genetic algorithm to detect epistatic interactions. Aflakparast et al. [15] presented an evolutionary-based heuristic search method CSE (Cuckoo Search Epistasis) to detect SNP interactions. CSE integrates the evolutionary-based optimization algorithm Cuckoo with the Bayesian network to mine SNP interactions. Tuo et al. [13] presented FHSA-SED, which adopts a harmony search algorithm with the Bayesian network and Gini-score to detect epistatic interactions.

Heuristic search has become a popular search strategy of epistatic interactions for its heuristic positive feedback and small search space for the past decades. However, heuristic search sometimes may lose the global optimum solutions for its approximate search strategy.

In recent years, Gene Expression Programming (GEP) algorithm is a notable evolutionary algorithm, which is a generalized method of Genetic Algorithm (GA) and Genetic Programming (GP) [33]. It has advantages for simply encoding complex problems and searching for global optimum solutions, and discovering rules and formulas [33–35]. Therefore, GEP algorithm has been widely adopted in solving complex nonlinear problems that are difficult to be solved by traditional methods for the possible loss of global optimum solutions [36–38].

**Fig. 1:**
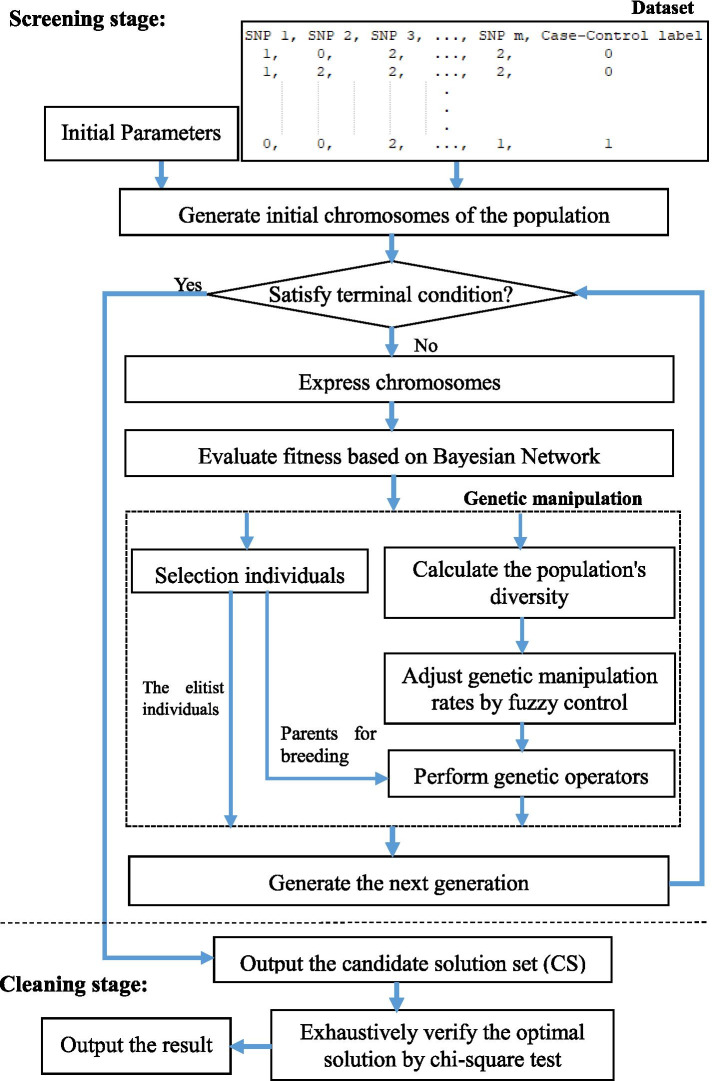
The framework of GEP-EpiSeeker

Motivated by GEP, we propose a novel evolutionary framework based on the GEP algorithm called GEP-EpiSeeker to detect epistatic interactions. Distinguishing from other evolutionary-based methods, GEP-EpiSeeker contains the screening and cleaning stages to find the SNP interactions associated with specific diseases. In the screening stage, we developed a tailor-made Gene Expression Programming algorithm named EpiGEP for screening suspected SNP interactions. In the cleaning stage, we conducted Chi-square tests for each screened SNP combinations produced by EpiGEP to identify the significant epistatic interactions. Fig. 1 summarizes the flowchart of the GEP-EpiSeeker.

## Results and discussion

We conducted experiments on 22 simulated disease models containing 12 disease models with marginal effects (DME) and 10 disease models with no marginal effects (DNME) to investigate the performance of GEP-EpiSeeker. The experimental results of GEP-EpiSeeker were compared with the experimental results gained from five state-of-the-art epistasis detection methods including BEAM [27], BOOST [24], AntEpiSeeker [30], MACOED [3] and EACO [14] in terms of power, recall, precision, and *F*1-score. Furthermore, we investigated the influence of the proposed fuzzy adaptive genetic manipulation rate on GEP-EpiSeeker performance. The simulation datasets for the 22 disease models, evaluation metrics, and parameter setting are introduced in the Methods section in detail.

### Comparison with state-of-the-art methods

**Fig. 2:**
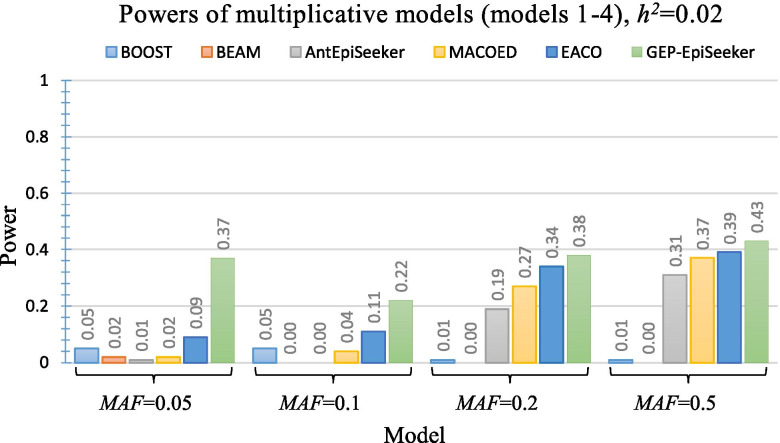
Power performance comparisons between GEP-EpiSeeker and other comparative methods on four multiplicative DME disease models

**Fig. 3:**
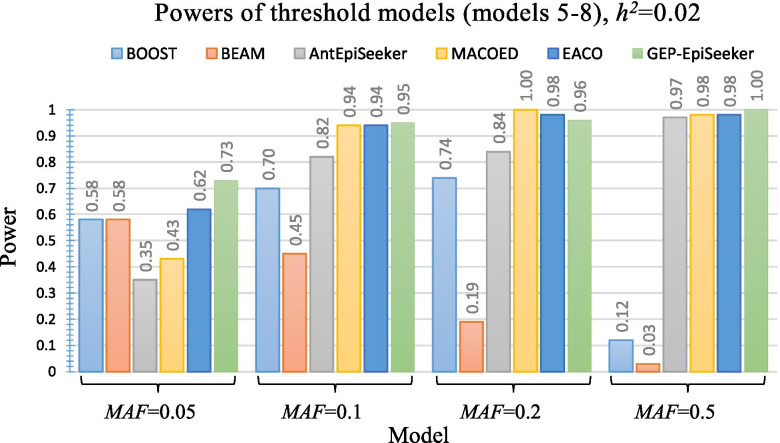
Power performance comparisons between GEP-EpiSeeker and other comparative methods on four threshold DME disease models

**Fig. 4:**
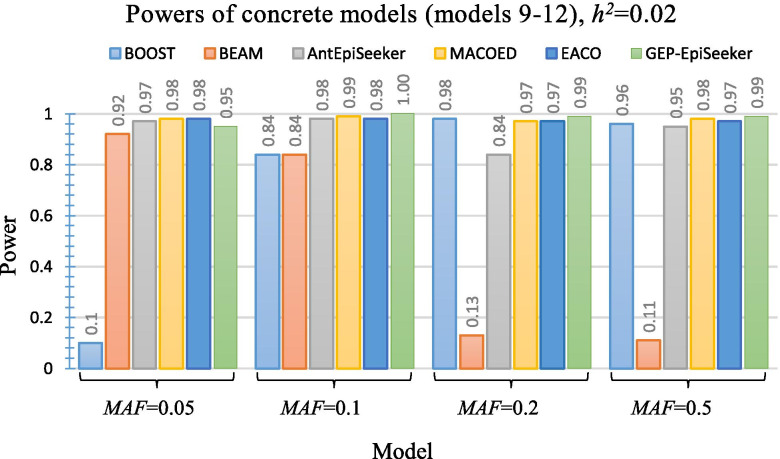
Power performance comparisons between GEP-EpiSeeker and other comparative methods on four concrete DME disease models

Figures 2, 3 and 4 present the performance of different methods on four multiplicative DME disease models (model 1 ~ model 4), four threshold DME disease models (model 5 ~ model 8) and four concrete DME disease models (model 9 ~ model 12), respectively. As shown in Fig. 2, GEP-EpiSeeker achieves higher power than all other methods and exhibits increasing power when *h*^2^=0.02. Similarly, as shown in Fig. 3 and Fig. 4, GEP-EpiSeeker outperforms all other methods in terms of power in most DME models with different parameter settings and is comparable with other methods in the rest DME models. Specifically, the power of GEP-EpiSeeker on the models 8 and 10 are equal to 1, and the power of GEP-EpiSeeker on the models 11 and 12 are equal to 0.99, due to the effective search guided by the chromosome evolution of GEP-EpiSeeker. These results indicate that the Bayesian fitness evaluation combined with the tailor-made chromosome evolution can fit the DME models well.

**Fig. 5:**
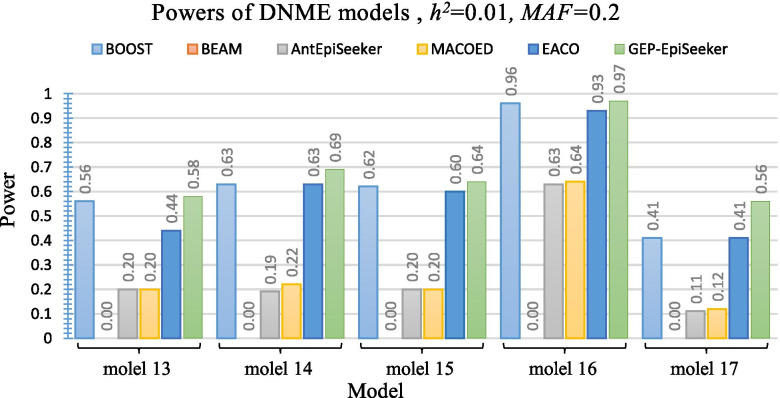
Power performance comparisons between GEP-EpiSeeker and other comparative methods on five different DNME disease models with *h*^2^=0.01 and *MAF*=0.2

**Fig. 6:**
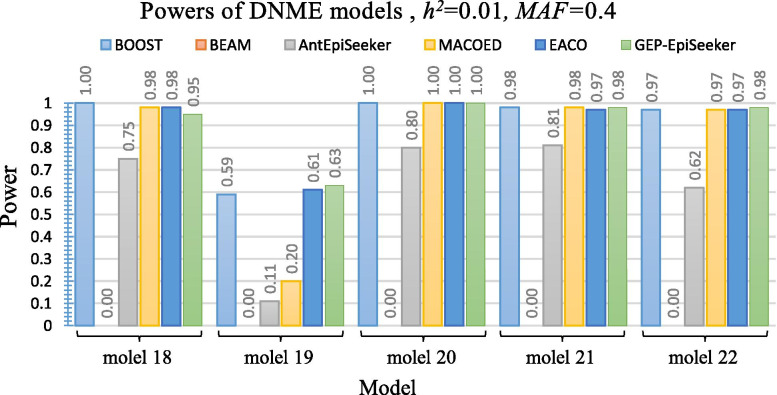
Power performance comparisons between the GEP-EpiSeeker and other comparative methods on the five different DNME disease models with *h*^2^=0.01 and *MAF*=0.2

Figure 5 and Fig. 6 present the performance of different methods on ten DNME disease models under *h*^2^=0.01 and *MAF*=0.2. The results of Fig. 5 and Fig. 6 reveal that GEP-EpiSeeker outperforms other methods on most DNME models. However, the power of GEP-EpiSeeker on DNME models does not reach the optimal level when comparing with its performance on DME models. This is because the SNP interactions in DNME models display no marginal effects and it is hard to capture these SNP interactions [14]. In addition, the performance of GEP-EpiSeeker are quite comparable with the performance of BOOST in most models, whereas the power of GEP-EpiSeeker is a little smaller than BOOST on DNME models 18 and 20. This is because the DNME models merely show interactive with no marginal effects whereas the mathematical model of BOOST only takes the interactive with no marginal effects into account, thus BOOST perfectly fits this dataset well.

To comprehensively evaluate the performance of our proposed method, we also compare the performance of the GEP-EpiSeeker and other methods in terms of recall, precision and *F*1 on all disease models. Tables 1, 2 and 3 show the comparison results of recall, precision and *F*1 on different disease models, respectively. Note that the values in brackets are the p-values of the t-test between results of GEP-EpiSeeker and the corresponding comparative method. As seen from Tables 1, 2 and 3, compared with other comparative methods, GEP-EpiSeeker achieves the best on 19 out of 22, 20 out of 22, and 17 out of 22 disease models in terms of recall, precision and *F*1, respectively. In terms of recall, GEP-EpiSeeker just slightly underperforms MACOED and EACO on the disease model 7, slightly underperforms EACO on the disease model 9, and has poor performance than MACOED, EACO, and BOOST on the disease model 18. In terms of precision, GEP-EpiSeeker just slightly underperforms MACOED on the disease models 6 and 17. In terms of F1, GEP-EpiSeeker just slightly underperforms MACOED and EACO on the disease models 6, 7, 9, 17, and 18. These results demonstrate that GEP-EpiSeeker outperforms comparative methods on most disease models. Overall, GEP-EpiSeeker is superior to state-of-the-art methods in the experiment. This indicates that the effective optimization of SNP combinations by the GEP algorithm greatly helps to narrow the search space and improve the power of our method. It is also interesting to see in Tables 1- 3 and Fig. 5 that the power, precision and *F*1 of the results produced by GEP-EpiSeeker achieves better performances than other comparative methods in most settings of DNME and DME models, demonstrating that the results of GEP-EpiSeeker on DNME and DME models are worth exploring, despite not obtaining correspondingly high levels.

**Table 1:** The recall of the comparative methods with different disease models

model id	BOOST	BEAM	AntEpiSeeker	MACOED	EACO	GEP-EpiSeeker
model 1	0.05±0.00 (0.00)	0.02±0.00 (0.00)	0.01±0.00 (0.00)	0.02±0.00 (0.00)	0.09±0.00 (0.00)	***0.37±0.03***
model 2	0.05±0.01 (0.00)	0±0.00 (0.00)	0±0.00 (0.00)	0.04±0.00 (0.00)	0.11±0.01 (0.00)	***0.22±0.02***
model 3	0.01±0.00 (0.00)	0±0.00 (0.00)	0.19±0.00 (0.00)	0.27±0.02 (0.00)	0.34±0.01 (0.00)	***0.38±0.01***
model 4	0.01±0.00 (0.00)	0±0.00 (0.00)	0.31±0.02 (0.00)	0.37±0.01 (0.00)	0.39±0.01 (0.00)	***0.43±0.01***
model 5	0.58±0.03 (0.00)	0.58±0.002 (0.00)	0.35±0.03 (0.00)	0.43±0.03 (0.00)	0.62±0.03 (0.00)	***0.73±0.01***
model 6	0.70±0.02 (0.00)	0.45±0.03 (0.00)	0.82±0.04 (0.00)	0.94±0.01 (0.00)	0.94±0.02 (0.03)	***0.95±0.01***
model 7	0.74±0.03 (0.00)	0.19±0.02 (0.00)	0.84±0.03 (0.00)	***1±0.00 (0.00)***	0.98±0.02 (0.03)	0.96±0.02
model 8	0.12±0.01 (0.00)	0.03±0.00 (0.00)	0.97±0.02 (0.00)	0.98±0.02 (0.00)	0.98±0.01 (0.00)	***1±0.00***
model 9	0.10±0.02 (0.00)	0.92±0.02 (0.00)	0.97±0.01 (0.00)	0.97±0.02 (0.00)	***0.98±0.02 (0.00)***	0.95±0.03
model 10	0.84±0.03 (0.00)	0.84±0.02 (0.00)	0.98±0.01 (0.00)	0.99±0.01 (0.00)	0.98±0.01 (0.00)	***1±0.00***
model 11	0.98±0.02 **(0.07)**	0.13±0.03 (0.00)	0.84±0.03 (0.00)	0.97±0.01 (0.00)	0.97±0.01 (0.00)	***0.99±0.01***
model 12	0.96±0.02 (0.00)	0.11±0.03 (0.00)	0.95±0.03 (0.00)	0.98±0.02 (0.05)	0.97±0.02 (0.00)	***0.99±0.01***
model 13	0.56±0.04 (0.00)	0±0.00 (0.00)	0.2±0.02 (0.00)	0.20±0.03 (0.00)	0.44±0.04 (0.00)	***0.58±0.03***
model 14	0.63±0.02 (0.00)	0±0.00 (0.00)	0.19±0.03 (0.00)	0.22±0.03 (0.00)	0.63±0.02 (0.00)	***0.69±0.02***
model 15	0.62±0.02 (0.02)	0±0.00 (0.00)	0.20±0.03 (0.00)	0.20±0.02 (0.00)	0.60±0.02 (0.00)	***0.64±0.02***
model 16	0.96±0.02 (0.12)	0±0.00 (0.00)	0.63±0.04 (0.00)	0.64±0.02 (0.00)	0.93±0.02 (0.00)	***0.97±0.01***
model 17	0.41±0.02 (0.00)	0±0.00 (0.00)	0.11±0.02 (0.00)	0.12±0.03 (0.00)	0.53±0.02 (0.00)	***0.56±0.02***
model 18	***1±0.00 (0.00)***	0±0.00 (0.00)	0.75±0.03 (0.00)	0.98±0.02 (0.00)	0.98±0.02 (0.00)	0.95±0.02
model 19	0.59±0.02 (0.00)	0±0.00 (0.00)	0.11±0.03 (0.00)	0.20±0.04 (0.00)	0.61±0.02 (0.00)	***0.63±0.01***
model 20	***1±0.00 (0.00)***	0±0.00 (0.00)	0.80±0.03 (0.00)	***1±0.00 (0.00)***	***1±0.00 (0.00)***	***1±0.00***
model 21	***0.98±0.02 (1.00)***	0±0.00 (0.00)	0.81±0.03 (0.00)	***0.98±0.02 (1.00)***	0.97±0.02 (0.00)	***0.98±0.01***
model 22	0.97±0.02 (0.02)	0±0.00 (0.00)	0.62±0.03 (0.00)	0.97±0.02 (0.03)	0.97±0.01 (0.00)	***0.98±0.01***

Note that the values in brackets are the *p*-values of the t-test between results of GEP-EpiSeeker and the corresponding comparative method. The best performances of each disease model are shown in bold and italics

**Table 2:** The precision of the comparative methods with different disease models

model id	BOOST	BEAM	AntEpiSeeker	MACOED	EACO	GEP-EpiSeeker
model 1	0.15±0.02 (0.00)	0.10±0.02 (0.00)	0.25±0.02 (0.00)	0.42±0.03 (0.00)	0.51±0.03 (0.00)	***0.62±0.03***
model 2	0±0.00 (0.00)	0.11±0.02 (0.00)	0±0.00 (0.00)	0.85±0.03 (0.01)	0.81±0.03 (0.00)	***0.87±0.02***
model 3	0±0.00 (0.00)	0.01±0.01 (0.00)	0.66±0.04 (0.00)	0.74±0.03 (0.00)	0.71±0.03 (0.00)	***0.78±0.03***
model 4	0±0.00 (0.00)	0.02±0.02 (0.00)	0.68±0.04 (0.00)	0.44±0.03 (0.00)	0.65±0.03 (0.00)	***0.72±0.03***
model 5	0.50±0.03 (0.00)	0.71±0.03 (0.00)	0.90±0.03 (0.00)	0.96±0.02 (0.00)	0.96±0.03 (0.01)	***0.98±0.01***
model 6	0.55±0.04 (0.00)	0.45±0.03 (0.00)	0.91±0.03 (0.00)	***0.98±0.02 (0.00)***	0.92±0.02 (0.00)	0.96±0.01
model 7	0.50±0.03 (0.00)	0.12±0.03 (0.00)	0.92±0.03 (0.00)	0.96±0.02 (0.11)	0.94±0.02 (0.00)	***0.97±0.01***
model 8	0.12±0.03 (0.00)	0.01±0.02 (0.00)	0.98±0.02 (1.00)	0.94±0.02 (0.00)	0.98±0.02 (1.00)	***0.98±0.01***
model 9	0.13±0.03 (0.00)	0.76±0.03 (0.00)	0.96±0.02 (0.00)	0.99±0.01 (0.01)	0.97±0.02 (0.00)	***1±0.00***
model 10	0.57±0.04 (0.00)	0.75±0.03 (0.00)	0.98±0.02 (0.04)	0.98±0.02 (0.04)	0.98±0.02 (0.04)	***0.99±0.01***
model 11	0.63±0.04 (0.00)	0.34±0.04 (0.00)	0.98±0.02 (0.04)	0.98±0.02 (0.04)	0.98±0.02 (0.04)	***0.99±0.01***
model 12	0.65±0.04 (0.00)	0.02±0.01 (0.00)	0.96±0.02 (0.00)	0.99±0.02 (0.05)	0.98±0.02 (0.00)	***1±0.00***
model 13	0.51±0.03 (0.00)	0±0.00 (0.00)	0.92±0.03 (0.00)	0.95±0.02 (0.04)	0.92±0.02 (0.00)	***0.96±0.01***
model 14	0.52±0.03 (0.00)	0±0.00 (0.00)	0.86±0.03 (0.00)	0.88±0.02 (0.00)	0.87±0.02 (0.00)	***0.91±0.02***
model 15	0.45±0.03 (0.00)	0±0.00 (0.00)	0.83±0.03 (0.00)	0.83±0.02 (0.00)	0.84±0.02 (0.00)	***0.87±0.02***
model 16	0.65±0.04 (0.00)	0±0.00 (0.00)	0.92±0.02 (0.00)	***0.99±0.02 (0.98)***	0.98±0.02 (0.04)	***0.99±0.01***
model 17	0.41±0.04 (0.00)	0±0.00 (0.00)	0.71±0.03 (0.00)	***0.75±0.03 (0.00)***	0.74±0.02 (0.00)	0.68±0.03
model 18	0.68±0.04 (0.00)	0±0.00 (0.00)	0.93±0.02 (0.00)	0.97±0.02 (0.05)	0.97±0.02 (0.05)	***0.98±0.01***
model 19	0.48±0.03 (0.00)	0±0.00 (0.00)	0.85±0.04 (0.00)	0.92±0.02 (0.00)	0.92±0.02 (0.00)	***0.94±0.02***
model 20	0.62±0.03 (0.00)	0±0.00 (0.00)	0.98±0.02 (0.00)	***1±0.00 (0.00)***	***1±0.00 (0.00)***	***1±0.00***
model 21	0.61±0.04 (0.00)	0±0.00 (0.00)	***0.98±0.02 (0.92)***	0.96±0.02 (0.00)	***0.98±0.02 (0.93)***	***0.98±0.01***
model 22	0.62±0.04 (0.00)	0±0.00 (0.00)	0.97±0.02 (0.04)	***0.98±0.02 (0.90)***	***0.98±0.02 (0.93)***	***0.98±0.01***

Note that the values in brackets are the *p*-values of the t-test between results of GEP-EpiSeeker and the corresponding comparative method. The best performances of each disease model are shown in bold and italics

**Table 3:** The *F*1 of the comparative methods with different disease models

model id	BOOST	BEAM	AntEpiSeeker	MACOED	EACO	GEP-EpiSeeker
model 1	0.08±0.02 (0.00)	0.03±0.02 (0.00)	0.02±0.03 (0.00)	0.04±0.02 (0.00)	0.15±0.03 (0.00)	***0.46±0.03***
model 2	0±0.00 (0.00)	0±0.00 (0.00)	0±0.00 (0.00)	0.08±0.02 (0.00)	0.19±0.04 (0.00)	***0.35±0.03***
model 3	0±0.00 (0.00)	0±0.00 (0.00)	0.30±0.04 (0.00)	0.40±0.04 (0.00)	0.46±0.03 (0.00)	***0.51±0.03***
model 4	0±0.00 (0.00)	0±0.00 (0.00)	0.43±0.04 (0.00)	0.40±0.03 (0.00)	0.49±0.03 (0.00)	***0.54±0.03***
model 5	0.54±0.03 (0.00)	0.64±0.02 (0.00)	0.50±0.04 (0.00)	0.59±0.03 (0.00)	0.75±0.04 (0.00)	***0.84±0.03***
model 6	0.62±0.03 (0.00)	0.45±0.02 (0.00)	0.86±0.02 (0.00)	***0.96±0.02 (0.04)***	0.93±0.03 (0.00)	0.95±0.02
model 7	0.60±0.04 (0.00)	0.15±0.02 (0.00)	0.88±0.03 (0.00)	***0.98±0.02 (0.00)***	0.96±0.02 (1.00)	0.96±0.02
model 8	0.12±0.04 (0.00)	0.02±0.02 (0.00)	0.97±0.03 (0.00)	0.96±0.02 (0.00)	0.98±0.02 (0.04)	***0.99±0.01***
model 9	0.11±0.04 (0.00)	0.83±0.03 (0.00)	0.96±0.03 (0.07)	***0.98±0.02 (0.03)***	0.97±0.02 (0.98)	0.97±0.01
model 10	0.68±0.04 (0.00)	0.79±0.03 (0.00)	0.98±0.02 (0.04)	0.98±0.02 (0.04)	0.98±0.02 (0.04)	***0.99±0.01***
model 11	0.77±0.03 (0.00)	0.19±0.03 (0.00)	0.90±0.03 (0.00)	0.97±0.02 (0.00)	0.97±0.02 (0.00)	***0.99±0.01***
model 12	0.78±0.03 (0.00)	0.03±0.02 (0.00)	0.95±0.02 (0.00)	0.98±0.02 (0.04)	0.97±0.03 (0.01)	***0.99±0.01***
model 13	0.53±0.03 (0.00)	0±0.00 (0.00)	0.33±0.04 (0.00)	0.33±0.03 (0.00)	0.60±0.04 (0.00)	***0.72±0.04***
model 14	0.57±0.03 (0.00)	0±0.00 (0.00)	0.31±0.05 (0.00)	0.35±0.04 (0.00)	0.73±0.03 (0.00)	***0.78±0.02***
model 15	0.52±0.04 (0.00)	0±0.00 (0.00)	0.32±0.04 (0.00)	0.32±0.04 (0.00)	0.70±0.04 (0.01)	***0.74±0.03***
model 16	0.78±0.03 (0.00)	0±0.00 (0.00)	0.75±0.02 (0.00)	0.78±0.04 (0.00)	0.95±0.02 (0.00)	***0.98±0.01***
model 17	0.41±0.05 (0.00)	0±0.00 (0.00)	0.19±0.04 (0.00)	0.21±0.04 (0.00)	***0.62±0.04 (0.45)***	0.61±0.04
model 18	0.81±0.03 (0.00)	0±0.00 (0.00)	0.83±0.03 (0.00)	***0.97±0.01 (0.02)***	***0.97±0.01 (0.02)***	0.96±0.01
model 19	0.53±0.04 (0.00)	0±0.00 (0.00)	0.19±0.04 (0.00)	0.33±0.04 (0.00)	0.73±0.03 (0.04)	***0.75±0.03***
model 20	0.77±0.03 (0.00)	0±0.00 (0.00)	0.88±0.05 (0.00)	***1±0.00 (0.00)***	***1±0.00 (0.00)***	***1±0.00***
model 21	0.75±0.03 (0.00)	0±0.00 (0.00)	0.89±0.03 (0.00)	0.97±0.01 (0.03)	0.97±0.02 (0.02)	***0.98±0.01***
model 22	0.76±0.03 (0.00)	0±0.00 (0.00)	0.76±0.03 (0.00)	0.97±0.01 (0.03)	0.97±0.01 (0.03)	***0.98±0.01***

Note that the values in brackets are the *p*-values of the t-test between results of GEP-EpiSeeker and the corresponding comparative method. The best performances of each disease model are shown in bold and italics

Although the recalls of MACOED and EACO on disease models 7, 9 and 18 are better than those of GEP-EpiSeeker, the precision of GEP-EpiSeeker is higher than that of MACOED and EACO. Similarly, the recalls of BOOST on models 18, 20 and 21 are higher or equal to those of GEP-EpiSeeker, but the precisions of GEP-EpiSeeker on these models are much higher than those of BOOST, thereby resulting in GEP-EpiSeeker’s superior performance in *F*1. These results demonstrate that GEP-EpiSeeker performs well in both recall and precision by coupling the EpiGEP algorithm with the Chi-square test.

Note that the values in brackets are the p-values of the t-test between results of GEP-EpiSeeker and the corresponding comparative method. The best performances of each disease model are shown in bold and italics.

### The influence of fuzzy adaptive genetic manipulation rate

**Fig. 7:**
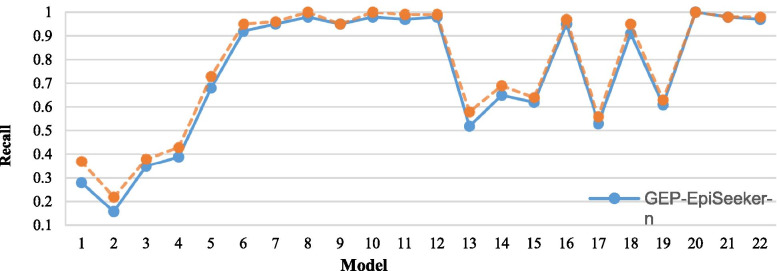
Comparison of GEP-EpiSeeker using the fuzzy adaptive genetic manipulation rate or not on the recall score

**Fig. 8:**
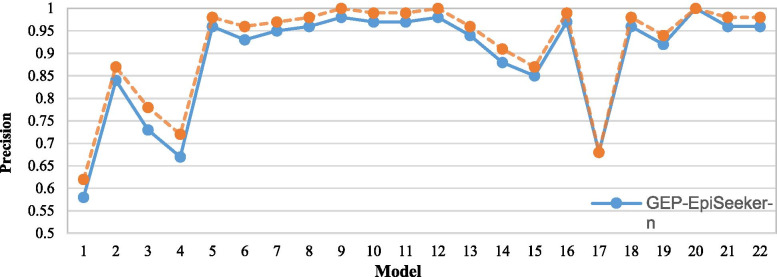
Comparison of GEP-EpiSeeker using the fuzzy adaptive genetic manipulation rate or not on the precision score

**Fig. 9:**
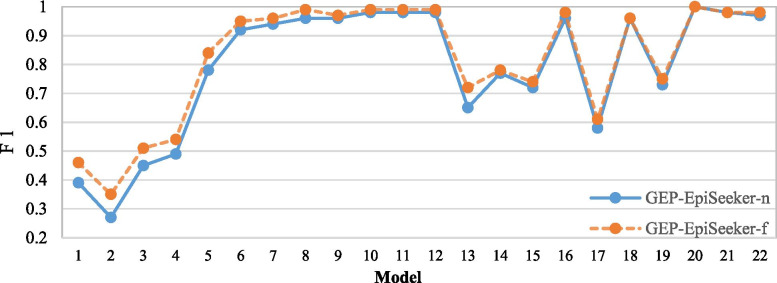
Comparison of GEP-EpiSeeker using the fuzzy adaptive genetic manipulation rate or not on the *F*1 score

In this section, we investigate whether fuzzy adaptive control will affect the performance of GEP-EpiSeeker. The comparisons on all metrics are based on the average score of 20 times of each epistasis model. Figures 7, 8 and 9 show the comparison result, where GEP-EpiSeeker-f represents that GEP-EpiSeeker uses fuzzy adaptive genetic manipulation rate, while GEP-EpiSeeker-n represents GEP-EpiSeeker does not use fuzzy adaptive genetic manipulation rate but uses the same fixed genetic manipulation rate as the original GEP. We observe from Figures 7- 9 that, on most epistasis models, GEP-EpiSeeker-f outperforms GEP-EpiSeeker-n over all the metrics. This indicates that the use of fuzzy adaptive genetic manipulation rate in GEP-EpiSeeker improves epistatic interaction detection, which is largely because the fuzzy adaptive genetic manipulation rate can improve the global search of GEP.

## Conclusion

In this work, we presented a novel method named GEP-EpiSeeker, based on the Gene Expression Programming algorithm, to identify epistatic interactions in Genome-wide Association Studies. In GEP-EpiSeeker, we proposed several tailor-made chromosome rules to depict SNP combinations, and integrated Bayesian network-based fitness function into the evolution of the chromosomes to search candidate SNP combinations and used the Chi-square test to identify optimal solutions from candidate SNP combinations.

Furthermore, we proposed two genetic operators with multiple and adjacent mutations and an adaptive genetic manipulation method with fuzzy control to improve the convergence and accuracy of our method. We conducted experiments on 22 disease models including 12 DME models and 10 DNME models to evaluate our method. Experimental results show that GEP-EpiSeeker is comparable or even superior to other comparative methods including BEAM, BOOST, AntEpiSeeker, MACOED and EACO in terms of power, recall, precision and *F*1-score on all datasets. These results indicate that GEP-EpiSeeker could be a promising alternative to the existing methods in epistasis detection and will provide a new way for accurately identifying epistasis.

Generally, the length of the GEP chromosome grows as the epistatic order increases, which results in a large increase in computation resources. A possible solution for this problem is to implement high-performance parallel algorithms for detecting epistasis interactions, which would be of interest in future work.

## Methods

For solving the epistasis detection problem with high dimension and small sample size, we transformed the identification of disease-causing SNP combinations into a heuristic combinatorial optimization problem. Then, GEP-EpiSeeker formulates SNP combinations using tailor-made GEP chromosome rules for epistasis detections, and discovers candidate SNP combinations by integrating Bayesian fitness evaluation with the tailor-made chromosome evolution, and finds optimal solutions from candidate SNP combinations by the Chi-square test. Furthermore, two genetic operators with multiple and adjacent mutations and an adaptive genetic manipulation method with fuzzy control are proposed to guide the tailor-made chromosome evolution, which helps to improve the convergence and accuracy of the algorithm.

In this section, we first briefly introduce the fundamentals of GEP in the first subsection. Then the proposed method GEP-EpiSeeker is introduced in detail, which involves the definitions of tailor-made chromosome and genetic operators, fuzzy adaptive control of genetic manipulation rate, and Bayesian network-based fitness function in the screening stage, and Chi-square tests for cleaning significant epistasis in the cleaning stage. Finally, we introduce the experimental method in this work, which involves the datasets, evaluation metrics for comparing the performance of the comparative methods, and the parameter setting.

### Fundamentals of Gene Expression Programming

Gene Expression Programming (GEP) is an excellent evolutionary algorithm, which is based on the gene expression law of biological genetics [33, 39]. GEP does not rely on gradient information and initial search point and is strong at searching optimum solutions [33, 39]. GEP heuristically searches the optimum solutions using chromosome evolution. A GEP chromosome consists of one or multiple genes. Each gene in the chromosome consists of a head and a tail. The head consists of function set *F*, which contains a series of simple functors, and terminator set *T*, which contains a series of decision variables and constants. The tail only consists of the terminator set. Assuming that the gene head length is *h*, the tail length *t* satisfies the following Exp. (1), where *n* is the maximum arity of the functors in *F*.

1 $$t=h\times \left(n-1\right)+1$$

**Fig. 10:**
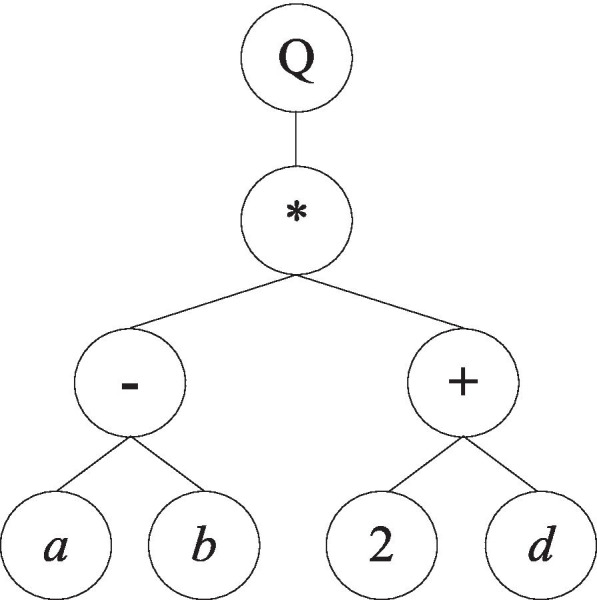
Example of GEP Expression Tree (ET)

The GEP chromosome has two forms of expression, one of which is the Karva expression (K-expression), and the other is the expression tree. Each gene in the chromosome can be expressed in a K-expression and an expression tree. Both K-expression and expression trees can be transformed into each other. We can transform the expression tree into K-expression by traversing the expression tree from top to bottom and left to right. Similarly, we can transform K-expression into an expression tree by filling the expression tree layer by layer with the symbols of K-expression from left to right. For example, Exp. (2) is a GEP chromosome with a gene of length 9, which includes functors {Q,*,-,+} and terminators {*a,b,c,d,*2},

2 $$\mathrm{Q}\ast -+ ab2 dc$$

where Q denotes the square-root function. According to GEP algorithm, the expression tree of the chromosome in Exp. (2) is shown in Fig. 10, and this expression tree can be interpreted as Exp. (3) in mathematics.

3 $$\sqrt{\left(a-b\right)\ast \left(2+d\right)}$$

**Fig. 11:**
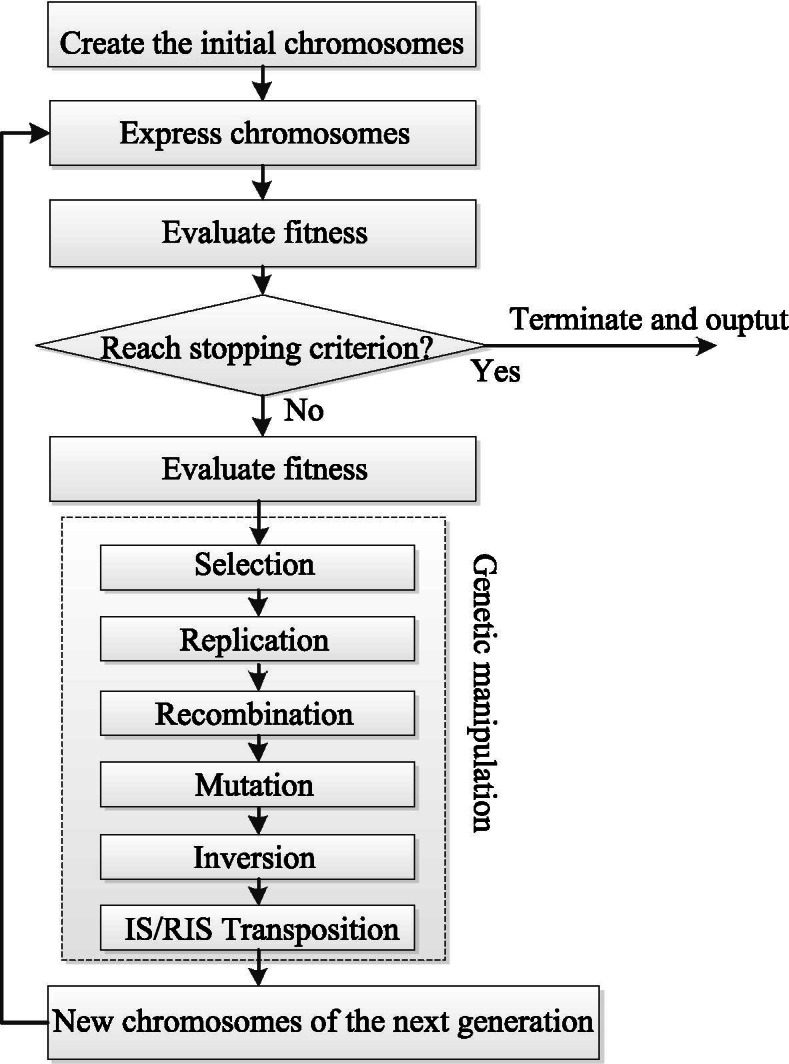
Flowchart of GEP algorithm

Each chromosome of GEP can be regarded as a solution of a target problem and is evaluated by the fitness function of GEP. The higher optimal fitness value of the solution is, the better solution represented in the chromosome is. The chromosomes can gradually evolve after a series of genetic manipulations until obtaining a solution with an acceptable fitness value. The genetic manipulation of GEP mainly includes selection, mutation, and crossover. The flowchart of GEP is shown in Fig. 11. For more details of GEP, please refer to [33, 36].

## Screening stage: EpiGEP for screening SNP combinations

**Fig. 12:**
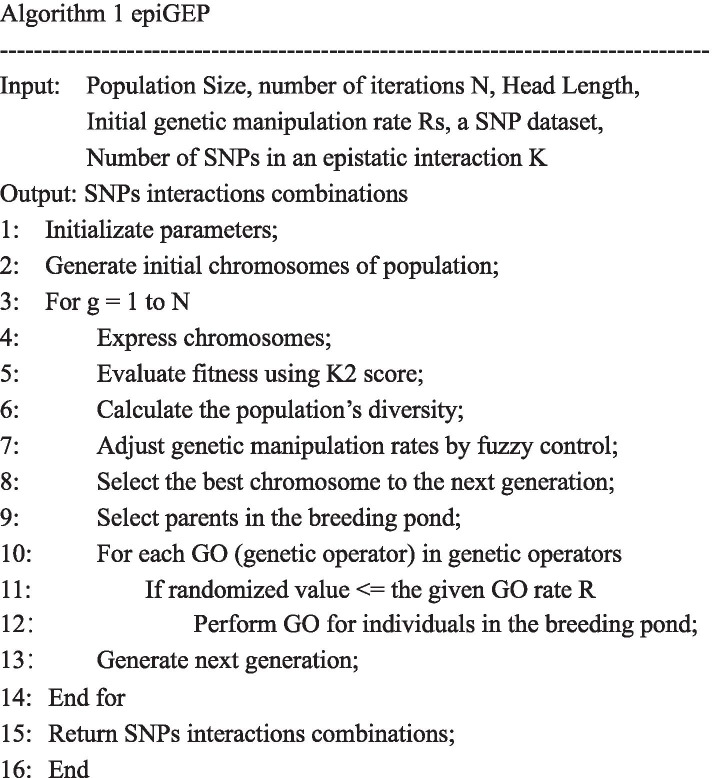
The pseudocode of EpiGEP

In this section, we will elaborate on our GEP-based algorithm named EpiGEP for detecting epistatic interactions. In EpiGEP, we proposed several tailor-made chromosome rules, two new genetic operators, and a tailor-made fitness function, and a genetic manipulation method with adaptive rate to accurately detect epistatic interactions. Fig. 12 provides the pseudocode of EpiGEP. In the following, we will elaborate on the procedure of EpiGEP.

### Tailor-made chromosome

In EpiGEP, each chromosome in a population is a candidate solution of a *k-way* SNP interaction combination that is associated with disease status *Y*. Recall that each gene in the GEP chromosome consists of a head and a tail. In EpiGEP, each gene consists of a head, a tail and an GT domain. The GT domain represents a genotype of one SNP. Let *Chr*_*i*_ be *i*th chromosome in a population with *L* chromosomes, *i=*1, 2, …, *L*. The chromosome *Chr*_*i*_ can be described by Exp. (4):

4 $$Chr_i=\left(S_{i1}\;Gt_{i1}\right)\;\left(S_{i2}\;Gt_{i2}\right)\;\left(S_{ij}\;Gt_{ij}\right)\dots$$

where *S*_*ij*_ is the *j*th SNP in the SNP dataset, *j=*1, 2…, *k*; *Gt*_*ij*_ is the variable of *S*_*ij*_ genotypes with values of {0,1,2}; (*S*_*ij*_*, Gt*_*ij*_) indicates a gene of the chromosome *Chr*_*i*_.

Exp. (5) gives an example of an EpiGEP chromosome *Chr*_*k*_. *Chr*_*k*_ is the *k*th chromosome with head length *h*=3 in a population, which is described as a 2-*way* SNP interaction combination with genotypes 0 and 2. In Exp. (5), *Chr*_*k*_ includes two genes: (*- + 1825, 0) and (+ *+ 3674, 2). In gene (*- + 1825, 0), the head is “*- +”, the tail is “1825” and the GT domain is “0”.

5 $$\left(\ast-+1825,\underline0\right)\left(+\ast+3674,\underline2\right)$$

In EpiGEP, any *k-way* (*k=*1, 2, 3, …) SNP interaction combination can be described by Exp. (4). In order to map each EpiGEP chromosome into a valid solution in SNP interaction detections, we define several idealized rules:

- EpiGEP only uses functors {+,-,*, /} and terminators {1, 2,…*, n*}, *n* is the total number of SNP in the dataset.
- Each chromosome in EpiGEP cannot contain identical SNP markers. The decoding result of *S*_*ix*_ and *S*_*iy*_ in a chromosome must not be identical (*x*≠*y*), or else this chromosome has to be mutated to get a new valid solution. The adjacent mutation is preferable (see section 3.2.2 for details).
- When EpiGEP decoding the expression trees of genes, the decoding results will be performed modulo by the number of SNP in the dataset. EpiGEP takes the absolute value of the modulo results as the final results.

**Fig. 13:**
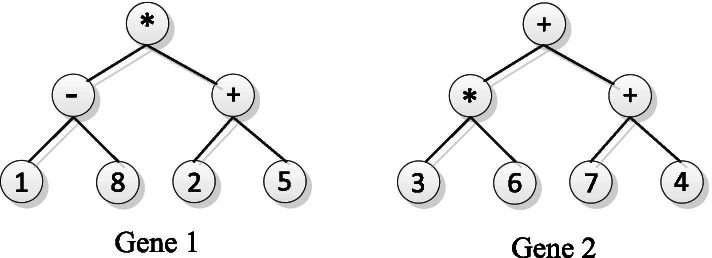
The expression trees of an EpiGEP chromosome

The EpiGEP chromosome of Exp.(5) can be encoded into expression trees and these trees are shown in Fig. 13. These expression trees can be decoded into 49 and 29, which correspond to the 49^th^ and 29^th^ loci of SNP, respectively. Then a candidate SNP interaction combination of *S*_*i49*_ and *S*_*i29*_ can be derived from the decoding results of these expression trees.

### Tailor-made genetic operators

EpiGEP inherits the genetic operators of GEP and expands two new genetic operators including adjacent mutation and multi-gene mutation to improve epistatic interaction detection. There are considerable correlations among neighboring SNPs in the genome as measure by linkage disequilibrium (LD) [15]. This is a helpful clue for finding epistatic interactions. We developed a novel genetic operator called adjacent mutation using the LD-specific heuristics to narrow the combination space and accelerate the convergence of EpiGEP. The adjacent mutation obeys the following idealized rules:

- The adjacent mutation performs mutation when a random number between 0 and 1 is smaller than the given threshold value called adjacent mutation rate;
- The adjacent mutation aims at refining the solutions with the neighborhoods of the current solution. To achieve this goal, the adjacent mutation only mutates at the tail or the GT domain of the objective gene. When adjacent mutation takes at the tail, the adjacent mutation randomly replaces the locus of the mutation point with one of the neighboring loci of the mutation point. When adjacent mutation takes at the GT domain, the adjacent mutation replaces the genotype with one of the rest genotypes.

In addition, we proposed another novel genetic operator Multi-gene mutation for EpiGEP. Multi-gene mutation simultaneously implements mutation operation on multiple points of different genes. The Multi-gene mutation could increase the diversity of population, assisting EpiGEP to jump out of the current search area, which avoids EpiGEP falling into local optimum to some extent and finally enhances the global exploration power of EpiGEP.

### Fuzzy adaptive control of genetic manipulation rate

The crossover rate of evolutionary algorithms will largely influence their convergence efficiency, while the mutation rate determines whether the algorithms can globally find the optimal solution out of the local optimum solution or not [40]. Nevertheless, similar to other evolutionary algorithms, GEP keeps the initial parameters unchanged during the procedure of the program. As evolution is ongoing, it is not easy to jump out of the local optimum solution due to the loss of population diversity.

In this work, we use a fuzzy control method to dynamically and automatically adjust the genetic manipulation rates of EpiGEP to find the globally optimum solution out of the local optimum solution.

First, population diversity is measured according to the dispersion degree of individual fitness in the population. Population diversity is evaluated by the ratio *d* of optimal fitness (*F*_*best*_) to average fitness (*F*_*ave*_) of the current population. Equation (6) is used to determine the population diversity when *F*_*best*_ ≤*F*_*ave*_. On the contrary, Equation (7) is used. As the population converges, *d* gradually approaches one.

6 $$d=\frac{F_{min}}{F_{ave}},{F}_{best}\le {F}_{ave}$$

7 $$d=\frac{F_{ave}}{F_{max}},{F}_{best}>{F}_{ave}$$

**Fig. 14:**
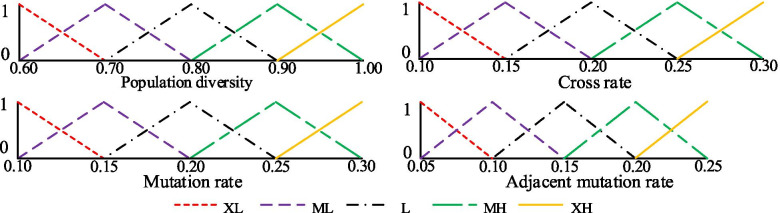
Input and output of the membership function

We designed some different fuzzy controllers to describe the size of population diversity and dynamically adjust the genetic manipulation rate. To simplify, we introduce how to use three different fuzzy controllers to adjust the crossover rate, mutation rate and adjacent mutation rate combined with fuzzy mathematics. These three fuzzy controllers use the current population diversity and the number of the current iterations as input. Outputs of the three fuzzy controllers are crossover rate, mutation rate and adjacent mutation rate of the next-generation population. Membership function of input and output is constructed by the triangular membership function and trapezoid membership function. Five fuzzy linguistic variables {*XL, ML, M, MH, XH*} are represented by low, low-medium, medium, medium-high and high diversity, respectively. They are used to describe the five fuzzy membership functions, as shown in Fig. 14. When the population diversity becomes low, GEP will increase the mutation rate to enhance diversity. When the population diversity becomes too high, GEP will increase the crossover rate and reduce the mutation rate.

### Bayesian network-based fitness function

**Fig. 15:**
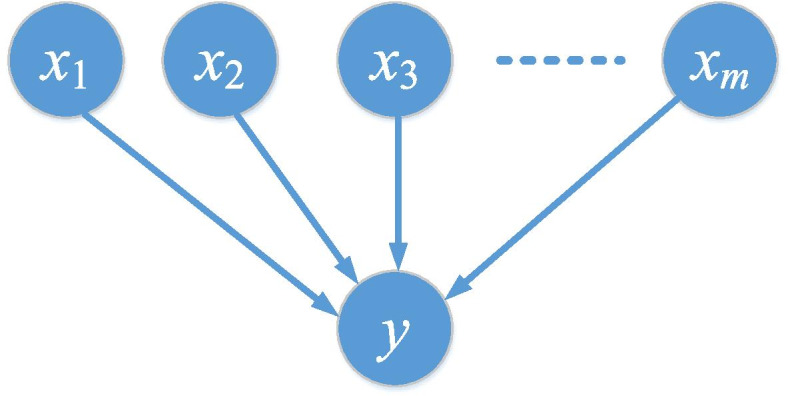
A *m*-SNP epistasis BN model between disease state *y* and *m* SNPs *x*_1_, *x*_2_, *x*_3_, . . . , *x*_*m*_

A Bayesian network (BN) is a probabilistic directed graphical model [3]. In the GWAS Bayesian network, a directed graphical BN model has consisted of a set of nodes and edges [6]. Each node represents a genotype or phenotype, while each edge represents the conditional dependencies between nodes. Given the Markov condition, in a BN model with *m*+1 nodes (*m* SNP nodes and a disease state), the joint probability distribution for the *m*+1 nodes can be calculated as the following [3, 6]:

8 $$p=\left({x}_1,{x}_2,\cdots, {x}_m\right)=\prod_{i=1}^mp\left({x}_i| pa\left({x}_i\right)\right)$$

where *pa*(*x*_*i*_) denotes the set of parent nodes of *x*_*i*_. An instance of *m*-SNP epistasis BN model is given in Fig. 15. Note that, in the epistasis BN model, there are only edges going from an SNP node to a disease node [6]. As we can see in Fig. 15, for an *m*-SNP epistasis BN model, the total number of combinations of SNP and disease state is ${C}_n^m$, where *n* is the total number of SNP in the SNP set.

In EpiGEP, we take the K2 score given in [3] as the fitness evaluation function. K2 score can be calculated as the following:

9 $$K2\ {score}_{log}=\sum_{i=1}^1\left(\sum_{b=1}^{r_i+1}\log (b)-\sum_{j=1}^J\sum_{d=1}^{r_{ij}}\log (d)\right)$$

where *I* is the total number of SNP combinations, and *I*=3^*m*^ as the possible values of SNP node are 0, 1 or 2. *J* denotes the state number of disease nodes [3]. *r*_*i*_ is the number of *i*th SNP combination and *r*_*ij*_ denotes the number of *i*th SNP combination connected with *j*th disease state [3, 6]. K2 score has been proposed to the *m*-locus epistasis detection in MACOED [3] and FHSA-SED [13], but these swarm intelligence based algorithms are only effective in detecting 2-locus epistasis. In this work, *m* can be set as a positive integer larger than 1 according to the users’ requirement.

## Cleaning stage: Chi-square tests for cleaning significant epistasis

In the screening stage, GEP-EpiSeeker gets a candidate solution set that consists of all suspected disease-causing SNP combinations. In the cleaning stage, the task of GEP-EpiSeeker is to identify the real disease-causing SNP combinations from candidate solutions. Previous researches [3, 41] showed that the Chi-square test can simply and powerfully identify the SNP combinations associated with the disease without considering disease models. GEP-EpiSeeker conducts an exhaustive search in candidate solutions with the Chi-square test to identify the significant epistasis. In the Chi-square test, the null hypothesis is that the candidate solution and the specific disease are not associated [3, 41]. The alternative hypothesis is that the candidate SNP combinations associated with the disease are accepted when the *P-*value of the Chi-square test is smaller than 0.05 [3, 41].

## Experimental method

### Datasets

We used 22 GWAS datasets corresponding to 22 epistasis models as GWAS datasets, which were generated by the classic simulation software GAMETES 2.0 [42]. GAMETES was widely used in the performance evaluation of epistasis detection [43]. In 22 epistasis models, there are 12 disease models with marginal effects (DME) and 10 disease models with no marginal effects (DNME).

The 12 DME models contain three types of DME epistasis models including 4 multiplicative models, 4 threshold models and 4 concrete models. These 12 DME models are produced by three different penetrance functions. These penetrance functions of the 12 DME epistasis models are shown in Table 4 [3]. These models have both marginal and interaction effects. The parameters *α* and *β* are used to control the penetrance table. The disease prevalence *P*(*D*), the genetic heritability *h*^*2*^ and the minor allele frequency *MAF* can be determined by *α* and *β* [3]. In this work, *P*(*D*)*=*0.1. In the experiments, the multiplicative models, threshold models and concrete models are named as model 1 ~ model 4, model 5 ~ model 8, model 9 ~ model 12, respectively.

**Table 4:** Penetrance functions of the three types of DME epistasis models

Multiplicative model		Loci 1		
		AA	Aa	aa
Loci 2	BB	*α*	*α*	*α*
	Bb	*α*	*α*(1+*β*)^2^	*α*(1+*β*)^3^
	bb	*α*	*α*(1+*β*)^3^	*α*(1+*β*)^4^
Threshold model		Loci 1		
		AA	Aa	aa
Loci 2	BB	*α*	*α*	*α*
	Bb	*α*	*α*(1+*β*)	α(1+*β*)
	bb	*α*	*α*(1+*β*)	*α*(1+*β*)
Concrete model		Loci 1		
		AA	Aa	aa
Loci 2	BB	*α*	*α*(1+*β*)	*α*(1+*β*)
	Bb	*α*(1*+β*)	*α*	*α*
	bb	*α*(1+*β*)	*α*	*α*

Note: The parameters {*α*, *β*} of the model 1~ model 12 are set as {0.0980, 0.7464}, {0.0960, 0.4329}, {0.0921, 0.2526}, {0.0782, 0.1610}, {0. 0958, 4.5647}, {0. 0918, 2.4771}, {0. 0836, 1.5108}, {0.0519, 1.6474}, {0.0804, 1.3856}, {0.0717, 1.2817}, {0.0608, 1.3997} and {0.0671, 1.3070}

The 10 DNME models (model 13 ~ model 22) are limited to the Hardy-Weinberg equilibrium (HWE) constraints but not limited to specific predetermined models. The penetrance table of the DNME models was produced by an exhaustive search.

Table 5 lists the details of 22 epistasis models. In each model of our experiments, there are 100 datasets with 750 controls and 750 cases genotyped by 100 SNPs.

**Table 5:** Penetrance tables of the twenty-two epistasis models with a different set of parameters

Model id	*h*^*2*^	*MAF*	Penetrance function			
			Genotypes (SNP *A*)	Genotypes (SNP *B*)		
				*BB*	*Bb*	*bb*
model 1	0.005	0.05	*AA*	0.0980	0.0980	0.0980
			*Aa*	0.0980	0.2989	0.5222
			*aa*	0.0980	0.5222	0.9121
model 2	0.005	0.1	*AA*	0.0960	0.0960	0.0960
			*Aa*	0.0960	0.1971	0.2824
			*aa*	0.0960	0.2824	0.4047
model 3	0.005	0.2	*AA*	0.0921	0.0921	0.0921
			*Aa*	0.0921	0.1445	0.1810
			*aa*	0.0921	0.1810	0.2266
model 4	0.005	0.5	*AA*	0.0782	0.0782	0.0782
			*Aa*	0.0782	0.1054	0.1223
			*aa*	0.0782	0.1223	0.1420
model 5	0.02	0.05	*AA*	0.0958	0.0958	0.0958
			*Aa*	0.0958	0.5331	0.5331
			*aa*	0.0958	0.5331	0.5331
model 6	0.02	0.1	*AA*	0.0918	0.0918	0.0918
			*Aa*	0.0918	0.3192	0.3192
			*aa*	0.0918	0.3192	0.3192
model 7	0.02	0.2	*AA*	0.0836	0.0836	0.0836
			*Aa*	0.0836	0.2099	0.2099
			*aa*	0.0836	0.2099	0.2099
model 8	0.02	0.5	*AA*	0.0519	0.0519	0.0519
			*Aa*	0.0519	0.1374	0.1374
			*aa*	0.0519	0.1374	0.1374
model 9	0.02	0.05	*AA*	0.0804	0.1918	0.1918
			*Aa*	0.1918	0.0804	0.0804
			*aa*	0.1918	0.0804	0.0804
model 10	0.02	0.1	*AA*	0.0717	0.1636	0.1636
			*Aa*	0.1636	0.0717	0.0717
			*aa*	0.1636	0.0717	0.0717
model 11	0.02	0.2	*AA*	0.0608	0.1459	0.1459
			*Aa*	0.1459	0.0608	0.0608
			*aa*	0.1459	0.0608	0.0608
model 12	0.02	0.5	*AA*	0.0671	0.1548	0.1548
			*Aa*	0.1548	0.0671	0.0671
			*aa*	0.1548	0.0671	0.0671
model 13	0.01	0.2	*AA*	0.6377	0.4884	0.3826
			*Aa*	0.4638	0.7645	0.9566
			*aa*	0.5798	0.5624	0.7189
model 14	0.01	0.2	*AA*	0.2216	0.2758	0.1414
			*Aa*	0.2587	0.1690	0.4013
			*aa*	0.2781	0.1279	0.4196
model 15	0.01	0.2	*AA*	0.2216	0.2758	0.1414
			*Aa*	0.2587	0.1690	0.4013
			*aa*	0.2781	0.1279	0.4196
model 16	0.01	0.2	*AA*	0.1391	0.1882	0.2214
			*Aa*	0.1901	0.1114	0.0198
			*aa*	0.2056	0.0514	0.2530
model 17	0.01	0.2	*AA*	0.1391	0.1882	0.2214
			*Aa*	0.1901	0.1114	0.0198
			*aa*	0.2056	0.0514	0.2530
model 18	0.01	0.4	*AA*	0.1032	0.0634	0.1242
			*Aa*	0.0978	0.0858	0.0693
			*aa*	0.0210	0.1467	0.0595
model 19	0.01	0.4	*AA*	0.1852	0.2908	0.2340
			*Aa*	0.2860	0.2009	0.2770
			*aa*	0.2486	0.2661	0.1657
model 20	0.01	0.4	*AA*	0.0731	0.0418	0.0146
			*Aa*	0.0240	0.0639	0.0591
			*aa*	0.0682	0.0188	0.0946
model 21	0.01	0.4	*AA*	0.0462	0.1275	0.0694
			*Aa*	0.1150	0.0667	0.0971
			*aa*	0.1067	0.0691	0.1085
model 22	0.01	0.4	*AA*	0.0950	0.1222	0.1267
			*Aa*	0.0973	0.1294	0.0999
			*aa*	0.2014	0.0439	0.1222

### Evaluation method

In this section, we compare the performance of GEP-EpiSeeker with other representative methods [3, 14, 24, 27, 30]. Following [3], we also used four common metrics including power, recall, precision and *F*1-score (*F*1) to evaluate the performance of these comparative methods. These metrics are defined as follows:

$$\mathrm{Power}=\frac{N_s}{N_d},$$

$$\mathrm{Recall}=\frac{TP}{TP+ FN},$$

$$F1=\frac{2\bullet Recall\ast Precision}{Recall+ Precision}$$

where *N*_*s*_ is the number of identified disease-causing models from all *N*_*d*_ datasets (in the experiments, *N*_*d*_= 100 for each disease model). *TP* denotes the number of SNP combinations associated with disease verified by the comparative algorithm, where the *P*-value of the Chi-square test is smaller than the given threshold (*P*<0.05). *FN* denotes the number of SNP combinations that are truly associated with disease but are identified as not associated with disease by the algorithm. *FP* denotes the number of SNP combinations that are not associated with disease but are identified as disease-related by the algorithm.

For each epistasis model and comparative method, it was independently run 20 times with the same 100 data files in our experiment to avoid stochastic deviation. For each epistasis model, we conducted some t-tests on 20 results of each method to validate the performance of the comparative models and GEP-EpiSeeker.

### Parameter setting

Since the Elitism mechanism can guarantee the global convergence of GEP [34], EpiGEP uses the roulette wheel selection model and the Elitism mechanism when it produces offspring. The parameters of GEP-EpiSeeker are: *population_size*=100, *number_of_iteration*=1000, *head_length*=5, i*nitial_genetic_manipulation_rate* = 0.3. EpiGEP will terminate when the number of iteration *N_i* >1000. For a *k*-locus epistasis detection, the number of gene *N_g* in an EpiGEP chromosome is *k*.

The parameters for BEAM and BOOST were set as the default of the BEAM and BOOST packages, respectively. Due to AntEpiSeeker, EACO and MACOED being three ACO-based methods, the parameter settings of these ACO-based methods were the same to conduct a fair comparison. The ant number and iteration number were set to 200 and 1000, respectively; the initial pheromone τ0 was set to 100; the parameters and that determine the weights of pheromone and heuristic information were set to 1. The evaporation coefficient of pheromones was set to 0.3. In addition, the rest of parameter settings for AntEpiSeeker were: largesetsize = 6, smallsetsize = 3, iItCountLarge = 150, iItCountSmall = 300.

## Data Availability

The Simulation datasets of the models were generated by GAMETES 2.0, that is available from https://surveillance.cancer.gov/genetic-simulation-resources/packages/gametes/. All other data that support the results of this study are available from the corresponding author upon request.

## Abbreviations

SNP: Single nucleotide polymorphism
GWAS: Genome-wide association studies
BEAM: Bayesian Epistasis Association Mapping
ACO: The ant colony optimization algorithm
MACOED: A multi-objective optimization heuristic method for identifying epistatic interactions
FAACOSE: A multi-objective ACO-based method for identifying epistatic interactions
EACO: An ACO-based method for identifying epistatic interactions by incorporating heuristic information multi-SURF into ant-decision rules
SURF: Spatially Uniform ReliefF
GE: A Genetic Algorithm-based hybrid algorithm, which is named genetic ensemble
CSE: A Cuckoo Search method for identifying SNP interactions
FHSA-SED: A harmony search algorithm with the Bayesian network and Gini-score for identifying epistatic interactions
GEP: The Gene Expression Programming algorithm
GA: The Genetic Algorithm
GP: The Genetic Programming algorithm
DME: Disease models with marginal effects
DNME: Disease models with no marginal effects
GT domain: A substructure of the chromosome that represents the gene type
LD: Linkage disequilibrium
BN: The Bayesian network
GAMETES: A simulation software for generating simulation GWAS datasets
MAF: The minor allele frequency
HWE constraints: The Hardy-Weinberg equilibrium constraints
TP: True Positive
TN: True Negative
FN: False Negative
FP: False Positive
